# Phenolic Characterization Using cLC-DAD Analysis and Evaluation of In Vitro and In Vivo Pharmacological Activities of *Ruta tuberculata* Forssk

**DOI:** 10.3390/antiox11071351

**Published:** 2022-07-11

**Authors:** Asma Saidi, Leila Hambaba, Mohamed Sabri Bensaad, Mohamed Akram Melakhessou, Chawki Bensouici, Nouicer Ferhat, Mohamed Amine Kahoul, Mahmoud Helal, Rokayya Sami, Saif A. Alharthy, Roua S. Baty, Nouf H. Alsubhi, Ghadeer I. Alrefaei, Abeer Elhakem, Sarah Alharthi, Fahmy G. Elsaid, Ali A. Shati

**Affiliations:** 1 Laboratory of Biotechnology of Bioactive Molecules and Cellular Physiopathology (LBMBPC), Department of Microbiology and Biochemistry, Faculty of Natural and Life Sciences, University Batna 2, Batna 05078, Algeria; asma.saidi@univ-biskra.dz (A.S.); l.hambaba@univ-batna2.dz (L.H.); m.bensaad@univ-batna2.dz (M.S.B.); m.melakhessou@univ-batna2.dz (M.A.M.); 2 Department of Natural Sciences and Life, Faculty of Exact Sciences and Natural Sciences and Life, Mohamed Khider University, Biskra 07000, Algeria; 3 Laboratory of Cellular and Molecular Physio-Toxicology-Pathology and Biomolecules (LPTPCMB), Department of Biology of Organisms, Faculty of Natural and Life Sciences, University Batna 2, Batna 05078, Algeria; 4 Biotechnology Research Center, Ali Mendjli UV 03, Constantine 25000, Algeria; c.bensouici@crbt.dz; 5 Laboratory of Environment, Health and Animal Production (LESPA), Institute of Agriculture and Veterinary Sciences, University Batna 1, Batna 05000, Algeria; ferhatimane@yahoo.fr; 6 Laboratory of Food Sciences (LSA), Institute of Agriculture and Veterinary Sciences, University Batna 1, Batna 05000, Algeria; aminekahoul@gmail.com; 7 Department of Mechanical Engineering, Faculty of Engineering, Taif University, P.O. Box 11099, Taif 21944, Saudi Arabia; mo.helal@tu.edu.sa; 8 Department of Food Science and Nutrition, College of Sciences, Taif University, P.O. Box 11099, Taif 21944, Saudi Arabia; 9 Department of Medical Laboratory Sciences, Faculty of Applied Medical Sciences, King Abdulaziz University, P.O. Box 80216, Jeddah 21589, Saudi Arabia; saalharthy@kau.edu.sa; 10 King Fahd Medical Research Center, King Abdulaziz University, P.O. Box 80216, Jeddah 21589, Saudi Arabia; 11 Department of Biotechnology, College of Science, Taif University, P.O. Box 11099, Taif 21944, Saudi Arabia; rsbaty@tu.edu.sa; 12 Biological Sciences Department, College of Science and Arts, King Abdulaziz University, Rabigh 21911, Saudi Arabia; nhalsubhi@kau.edu.sa; 13 Department of Biology, College of Science, University of Jeddah, P.O. Box 80327, Jeddah 21589, Saudi Arabia; galrefaei@uj.edu.sa; 14 Department of Biology, College of Science and Humanities in Al-Kharj, Prince Sattam Bin Abdulaziz University, Al-Kharj 11942, Saudi Arabia; a.elhakem@psau.edu.sa; 15 Department of Chemistry, College of Science, Taif University, P.O. Box 11099, Taif 21944, Saudi Arabia; sarah.alharthi@tu.edu.sa; 16 Biology Department, Science College, King Khalid University, P.O. Box. 960, Abha 61421, Saudi Arabia; felsaid@kku.edu.sa (F.G.E.); aaalshati@kku.edu.sa (A.A.S.); 17 Zoology Department, Faculty of Science, Mansoura University, Mansoura 35516, Egypt

**Keywords:** *Rutatuberculata*, antioxidant, anti-inflammatory, anti-ulcer, anti-cholinesterase, cLC-DAD analysis

## Abstract

The perennial aromatic plant *Ruta tuberculata* Forssk (Rutaceae) has been traditionally used by Mediterranean peoples as folk medicine against several types of disease to treat diverse illness. The objective of this work is to evaluate the in vitro and in vivo pharmacological activities of the aqueous (RAE) and methanolic (MeOH) 80% (RME) extracts of Algerian *R. tuberculata* aerial parts. Antioxidant potential, neuro-protective and anti-arthritic activities were investigated in vitro using six antioxidant approaches and by determining acetyl-cholinesterase and bovine albumin denaturation inhibitory capacities, respectively. Furthermore, in vivo anti-ulcer and anti-inflammatory activities were evaluated on EtOH-induced gastric mucosal damage and carrageenan-induced paw edema models in mice. Moreover, bio-compounds’ contents were also quantified using spectrophotometric and cLC-DAD methods. Both in vivo and in vitro investigations showed remarkable antioxidant activity of *Ruta tuberculata* Forssk, while methanolic extract (RME) of *Ruta tuberculata* Forssk exhibited more significant neuro-protective and anti-inflammatory effects. However, the antiulcer activity was more pronounced with RAE of *R. tuberculata*, which suggests that this plant can be considered as a natural resource of potent bioactive compounds that may act as antioxidant and anti-inflammatory agents, which underlines the importance of incorporating them in therapies in order to treat various diseases linked to oxidative stress, and they may also provide crucial data for the development of new anticholinesterase drugs to improve neurodegenerative diseases, such as Alzheimer’s.

## 1. Introduction

Nowadays, several pharmacological studies affirmed that free radicals generated during oxidation processes could be involved in protein alteration, lipid peroxidation, DNA oxidation and even cell apoptosis, which can significantly contribute to the development of many disorders, in particular, cardiovascular, rheumatoid diseases, gastric ulcers and Alzheimer disorders [1,2,3]. Indeed, the inflammatory response, as a complex and vital immune defense mechanism, displays a crucial role in the alleviation of tissue damage during injuries, such as pro-inflammatory mediators including prostaglandins, histamine, serotonin and some key cytokines, in the resolution step of the inflammatory process in order to repair and regenerate damaged tissues [4,5]. Alzheimer’s is a progressive neurodegenerative disease that requires the use of treatments such as acetyl-cholinesterase inhibitor drugs (AChEIs), generally coupled with a high level of antioxidants and, occasionally, anti-inflammatory drugs to counter complications related to this pathology [1]. However, the use of synthetic AChEI drugs is undesirably perceived by consumers due to their side effects, such as hypatotoxicity, gastro-intestinal disturbances, vomiting and diarrhea [1]. Furthermore, non-steroidal anti-inflammatory drugs (NSAIDs) are given as complementary treatment, but may present some drawbacks when used excessively during a long period of time due to the acquisition of drug resistance phenomenon, which underlines the urgent situation to develop more effective natural anti-inflammatory drugs with fewer undesirable effects [6,7]. Recently, there is a growing interest in studying medicinal plants in order to understand their full pharmacological potential along with preventing the development of chronic and degenerative diseases. Indeed, plants are considered a natural source for desired secondary metabolites such as phenolic compounds, flavonoids, tannins, terpens, essential oils, alkaloids and other bioactive compounds. The presence of these bioactive compounds is responsible for the antioxidant activity of *Ruta tuberculata* Forssk, which helps in the prevention against several types of diseases including preventing the development of chronic illness related to oxidative stress by inhibiting or reducing free radicals’ production, such as ROS and NOS [8,9]. Furthermore, the anti-inflammatory, anti-ulcer [2,5], neuroprotective and anti-cholinesterase activities of these bio-compounds were widely screened and documented [10]. *Ruta tuberculata* Forssk (also called *Haplophyllum tuberculatum* (Forssk.) Adr. Jussi.), known as ‘El fijel’, is an aromatic endemic plant that originates from Mediterranean to eastern Siberian zones and belongs to the *Ruta* (*haplopylum*) genus [11]. The *Ruta* genus belongs to the Rutaceae family and includes more than 1800 species, characterized by green foliage with yellow flowers, which preferentially grow in tropical and temperate regions. Among *Ruta* species, *R. tuberculata* is incorporated in Mediterranean traditional medicine and often used in Algeria to treat skin pathologies, gastric, intestinal diseases, rheumatism, diabetes and hypertension [11,12], but also to treat various neurological illness such as hysteria, vertigo and epilepsies, noting that their infused leaves are prescribed to prevent convulsion and other nervous disorders [11]. In addition, several studies have reported the antioxidant, anti-inflammatory, anti-ulcer, hepatoprotective and anti-Alzheimer effects of *Ruta* plants [1,2,3,13]. In fact, the pharmacological proprieties of *Ruta* species could be attributed to the presence of diverse chemical classes of secondary biocompounds, including coumarine, alkaloid derivatives [8,14], essential oils [11,15], lignanes [16], polyphenols and flavonoids [3,17,18]. Based on the previous literature, there are no studies reported on phenolic characterization of the aerial parts of *R. tuberculata* growing in Algeria and their pharmacological activities. 

In this context, this work aimed to screen the phenolic compounds’ content in *R. tuberculata* aqueous and methanolic crude extracts, but also to investigate their antioxidant, neuroprotective, anti-ulcer and anti-inflammatory properties.

## 2. Materials and Methods

### 2.1. Chemicals

All analytical grades of dissolvent, reagents and pure phenolic compounds such as Methanol, Folin–Ciocalteu Reagent, sodium carbonate (Na_2_CO_3_), Aluminium Trichloride (AlCl_3_), Hydrochloric Acid (HCl), Gallic Acid (C_7_H_6_O_5_), Catechin (C_15_H_14_O_6_), Quercetin (C_15_H_10_O_7_), Vanillin (C_8_H_8_O_3_), Sodium Phosphate Monobasic (NaH_2_PO_4_), Sodium Dibasic Phosphate (Na_2_HPO_4_), Sulfuric Acid (H_2_SO_4_), Potassium persulfate (K_2_S_2_O_8_), Iron chloride (II) (FeCl_2_), Iron Trichloride (FeCl_3_), Ammonium acetate, Sodium hydroxide (NaOH), Potassium Hydroxide (KOH), 1,1-Diphenyl-2-picryl- hydrazyl (DPPH), β-carotene, Linoleic Acid, Tween 40, Trichlo-roacetic acid (TCA), Potassium ferricyanide (K_3_Fe[CN]_6_), Ammonium molybdate (NH_4_) _2_MoO_4_, Acetylcholinesterase (AChE) type VI-S from electric eel < 1000 U/mg solid, Acetylcholine iodide (ACI), 5,50-dithiobis (2-nitrobenzoic acid) (DTNB), Carragenean, Indometacin, Omepazole, Galanthamine, Butylated hydroxytoluene BHT (C_15_H_24_O), Butylate hydroxyanisole BHA (C_11_H_16_O_2_) and Ascorbic Acid (C_6_H_8_O_6_) were purchased from Sigma-Aldrich and Fluka.

### 2.2. Plant Material and Extraction

#### *Ruta tuberculata* 

Forssk was collected in March 2017 from the agricultural area of Rase El-miaade (Ouled Djellal, Algeria). The specimen was identified by Dr. Salem-Court N. (Voucher specimen no: Fl.AA 86-86-1775/TRO-28101397)

The aqueous extract (RAE) of the plant was prepared from aerial parts by the decoction method in ratio 1:10 (*w*:*v*) [19], while the MeOH–H_2_0 extract (RME) was prepared by the cold maceration method [20]. The prepared mixture was filtered to obtain a concentrated transparent extract and then stored at −4 °C for further analysis. The yield of extract in order to different solvents was calculated using the following formula: % Yield = (Weight of dry extract/Weight of taken plant for extraction) × 100. Greenish powders were used with the respective extraction yield values of 19.8 ± 2.3 and 11.3 ± 2.2%.

### 2.3. Phytochemical Analysis

#### 2.3.1. Determination of Total Bioactive Compounds’ Content

Total polyphenols content (TPC) was estimated according to Folin–Ciocalteu’s approach [21]. The absorbance was measured at 765 nm, and TPC was calculated using the calibration curve of gallic acid (20–160 µg/mL). Data were expressed as mg gallic acid equivalent (mg GAE/g dried extract).

Total flavonoid content (TFC) was determined using aluminum chloride reagent, as previously explained by Bahorun et al. [22]. The absorbance was measured at 430 nm and the total flavonoid content was estimated through a standard curve. Data were presented as mg quercetin equivalent (mg QE/g dried extract).

Condensed tannins content (CTC) was assessed based on the spectrophotometric method developed by Heimler et al. [23]. The absorbance of the mixtures was measured at 500 nm. The condensed tannins amount was calculated using the calibration curve of catechin (20–200 µg/mL) as a standard.

#### 2.3.2. cLC-DAD Analysis

The studied extracts were submitted in the present work to the cLC-DAD quantitative analysis, whereas the use of the DAD detection system could allow us to bring new compositional data about the contained polyphenols in *R. tuberculata* aerial parts.

The different plant extracts were further analyzed using an Agilent cLC instrument mod. 1260 infinity series (Agilent Technologies, Waldbronn, Germany) coupled with a G1311B quaternary capillary pump, a degasser and G4212B diode array detector. The capillary analytical column used for the analysis was a Thermo ODS Hypersil 250 × 4.6 mm, 5 µm column, which was preserved at room temperature and equipped with an autosampler (1329 B) and an injection valve coupled with an external stainless-steel loop. Polyphenols were detected at multi-wavelength ranges of 254 nm, 280 nm, 325 nm and 360 nm, respectively, using a DAD (G4212 B)-UV/Vis spectrophotometer (Agilent Technologies) connected to original bundle PC chemstation software (Agilent Technologies, Germany), which was employed to acquire and analyze data. The chromatographic analysis of the corresponding extracts was performed according to Erenler et al.’s method [24], using a mobile phase included eluent A (0.1% formic acid in water) and eluent B methanol. The flow rate was adjusted at 0.7 mL/min at 40 °C with an injection volume of 20 µL. The *R*. *tuberculata* extracts and standards were solubilized in a methanol (HPLC grade) at a concentration of 1 mg/mL and then filtrated through a cellulose filter membrane (part no. 3150-0576, 47 mm, pore size 0.45 µm, 100/pk, Germany). Detected polyphenols in different extracts were identified based on their retention time and UV absorption, compared to that recorded by the pure standards, until which they were quantified at their maximum absorption wavelength: 254 nm for p-hydroxybenzoic acid; 280 nm for gallic acid, catechin, vanillic acid, p-coumaric acid, cinnamic acid, trans-cinnamic acid, naringenin and sylimarin; 325 nm for caffeic acid and trans-ferulic acid; and 360 nm for rutin, myricetin and quercetin.

### 2.4. In Vitro Antioxidant Activity

#### 2.4.1. DPPH Free Radical-Scavenging Capacity

In terms of hydrogen donating ability, the radical-scavenging activity of *R. tuberculata* polar extracts was evaluated by the standard DPPH (1,1-Diphenyl-2-picryl- hydrazyl) method followed by Blois [25]. The purple colored DPPH solution turns yellow-colored when it is reduced by an antioxidant scavenger of radical. Briefly, 40 μL of various dilution samples, extract or synthetic antioxidants such as BHA, BHT, ascorbic acid and pure phenolic compounds (gallic acid and quercetin) were mixed with a 160 μL of methanol DPPH solution (0.004%). The reaction mixture was shaken and kept at room temperature in the dark for 30 min. The absorbance of the samples was read at 517 nm using EnSpire^®^ multimode plate reader (PerkinElmer, Waltham, MA, USA). The results were expressed as the concentration corresponding to the inhibition of 50% of the DPPH radical (IC_50_), which was determined from the plotting of the inhibition’s percentage of DPPH that was calculated using the following equation (Equation (1)):

I (%) = [(A_control_ − A_sample_)/A_control_] × 100 (1)

A_control_ is the absorbance of blank and the A_sample_ is that of extract or standard sample.

#### 2.4.2. ABTS Radical Cation Scavenging Activity

The scavenging activity of *R. tuberculata* crude extracts (AcE and EtOAcE) was determined spectrophotometrically at different concentrations against the ABTS cation radical according to the previous method [26]. ABTS^+^ solution was produced by mixing 2 mM of ABTS in H_2_O with 2.45 mM potassium persulfate (K_2_S_2_O_8_). This solution was stored in the dark for 16 h, then it was diluted to provide an absorbance where 0.700 ± 0.02. 160 µL of the ABTS cation solution was added to 40 µL of each sample of extracts or reference drugs (BHT and BHA). Samples were then kept in the dark for 6 min and after 30 min of incubation. Thereafter, the absorbance of the samples was recorded at 734 nm (PerkinElmer EnSpire^®^, Pleasanton, CA, USA), and the inhibition percentage was calculated using Equation (1).

#### 2.4.3. Total Antioxidant Capacity by Phosphomolybdenum Assay (TAC)

This approach was estimated using molybdate ion reduction as described by Prieto et al. [27], based on the formation of a green phosphate/Mo (V) complex at acid pH when the Mo (VI) was reduced to Mo (V) by the sample. Next, 100 μL of each extract and 1 mL of reagent solution (0.6 M sulfuric acid, 28 mM sodium phosphate and 4 mM ammonium molybdate) were incubated at 95 °C for 90 min. After cooling of the mixture at room temperature, the absorbance of each tube was read at 695 nm. The standard curve (0.0027x + 0.3535, R^2^ = 0.997) was prepared using different concentrations (10–300 µg/mL) of ascorbic acid as an antioxidant drug. The antioxidant capacity of extracts was expressed in terms of mg of ascorbic acid equivalent AAE/g of dry extract.

#### 2.4.4. Ferric Reducing Antioxidant Power (FRAP) Assay

FRAP activity of the *R. tuberculata* extracts was determined using a method followed by Oyaizu [28], with minor modifications, and the capacity of *R. tuberculata* extracts to reduce the Fe^+3^ ferric irons to the ferrous irons Fe^+2^ irons was examined. The increased absorbance, which was measured at 700 nm (PerkinElmer EnSpire^®^, USA), signified the powered reducing activity. A volume of 40 µL of sodium phosphate buffer (0.2 M, pH 6.6) and 50 µL of 1% potassium ferricyanide (K_3_Fe (CN)_6_) was added to 10 µL of different concentrations of each extract or standard, using BHT, BHA, quercetin, gallic and ascorbic acids as positive controls. The reaction mixture was incubated for 20 min at 50 °C. A volume of 50 µL of trichloroacetic acid (10%) was added to acidify the mixture. Then, 40 µL of distilled water and 10 µL of FeCl_3_ (0.1%) were added to the mixture before the absorbance reading. The results were expressed as the concentration (µg/mL) corresponding to the absorbance value 0.50 (A_0.5_) at 700 nm.

#### 2.4.5. Cupric Reducing Antioxidant Capacity (CUPRAC)

The Cu^+2^ ion reducing capacity of the tested extracts was assessed using the CUPRAC approach [29], based on the reduction of Copper (II) to copper (I) in the presence of neocuprine for giving a stable complex which has a maximal absorbance at 450 nm. Briefly, 50 μL of 10 mM copper (II) chloride solution (CuCl_2_ × 2H_2_O) were mixed with the same volume of neocuprine (Nc) and 60 μL of ammonium acetate (AcNH4) buffer solution (1 M, pH 7.0). Various concentrations of each extract or standard (BHT and BHA) were added to the initial mixture to obtain a final volume of 200 μL. After 1 h of incubation in the dark, absorbance was measured at 450 nm (PerkinElmer EnSpire^®^, USA). The results were calculated as A_0.5_ (µg/mL).

#### 2.4.6. β-Carotene–Linoleic Acid Bleaching Inhibition Assay

The potential antioxidant of *R. tuberculata* extracts to inhibit the lipid-peroxidation was evaluated by the measurement of the β-carotene bleaching, according to the method previously described by Marco [30], with slight modifications. BHT and BHA were used as antioxidant references. The β-carotene emulsion was prepared as follows: in a round-bottomed flask, 0.5 mg of the β-carotene was mixed to 200 mg of Tween 40 and 25 μL of linoleic acid dissolved in 1 mL of chloroform. Under vacuum, chloroform was eliminated and then 100 mL of H_2_O_2_ was added to the emulsion with vigorous shaking. Then the absorbance of the emulsion was adjusted to 0.8–0.9 at 470 nm. Next, 160 μL of the emulsion was added to 40 μL of corresponding extracts or standards at different concentrations. The reaction mixture was incubated up to 120 min at 50 °C. Absorbance of the mixtures was measured at 470 nm (PerkinElmer EnSpire^®^, USA) before and after the incubation rate. The percentage of the antioxidant activity (AA) was calculated using the following equation (Equation (2)):

AA (%) = [1 − (A_H0_ − A_Ht_/A_C0_ − A_Ct_) × 100] (2)

A_H0_: Absorbance in initial time (0) in presence of the sample; A_Ht_: Absorbance after 120 min in presence of the sample; A_C0_: Absorbance in initial time (0) in presence of the control; A_Ct_: Absorbance after 120 min in presence of the control.

### 2.5. Anti-Acetylcholinesterase Activity

Acetylcholinesterase (AChE) inhibitory activity of the extracts was carried out by the previous method of Ellman et al. [31], employing the acetylcholine iodide (ACI) as a substrate of the reaction and the Galantamine as a positive control. Then, 20 µL of AChE solution (5.32 × 10^−3^ U) was mixed with 150 µL of sodium phosphate buffer (0.1 M, pH 8.02) and 10 µL of each extract sample (3.125–200 µg/mL) or standard. The reaction mixture was incubated for 15 min at 37 °C. Thereafter, 10 µL of the revealing solution DTNB (5.50-Dithio-bis (2-nitrobenzoic) acid (0.5 mM)) and 10 µL of ACI (0.73 mM) were added. A blank was prepared by addition of sample solution to all reaction reagents without enzyme solution. Sample and blank absorbance were measured at 412 nm at 0 and 15 min. Blank absorbance was subtracted from that of the samples and the acetylcholinesterase inhibitory activity was calculated as follows (Equation (3)):

I (%) = 100 − [(A_Sample_ − A_Blank_)/A_Control_ × 100] (3)

### 2.6. Acute Oral Toxicity

Male *Swiss albino* mice (25–30 g) were purchased from the Pasteur Institute—Laboratory of Animals (Algiers, Algeria) and housed for 10 days to acclimatize with the standardized laboratory conditions (at 21–24 °C and a light/dark cycle of 12 h/12 h) with free access to classic food and water before the beginning of the experimentation. All manipulations performed on animals respected the criteria of the National Institute of Health guidelines [32] and were approved by the Biology Animal Ethics Committee of University of Batna-2, Algeria (approval n. 14/DBO/FSNV/UB2/2017).

According to the internationally acceptable guidelines N°425 of the Organization of Economic Cooperation and Development OECD [33], the acute oral toxicity of studied extracts was early performed at limit doses of 2000 and 5000 mg/kg. The behavioral changes and mortality were checked individually during a period of 14 days.

### 2.7. In Vitro and In Vivo Anti-Inflammatory Activities

#### 2.7.1. In Vitro Anti-Arthritic Activity (Bovine Albumin Denaturation Assay)

The anti-inflammatory activity of *R. tuberculata* polar extracts was investigated in vitro by the previous method of bovine serum albumin (BSA) denaturation [34]. Diclofenac sodium was incorporated as an anti-inflammatory drug (positive control). A reaction mixture containing 500 µL of BSA solution (0.2%) prepared in Tris-HCl Buffer Saline (pH 6.6) and 500 µL of different concentrations of each extract or diclofenac sodium (50–800 μg/mL) was incubated at 37 °C for 15 min and then heated at 70 °C for 5 min. The absorbances were measured at 660 nm after cooling of the reaction solutions at room temperature. For the control solution preparation, the test samples were replaced with double-distilled water and the inhibition percentage of the protein denaturation was calculated using Equation (1) (Equation (1)).

#### 2.7.2. Carrageenan-Induced Paw Edema

The anti-inflammatory effect of RAE and RME against the acute skin inflammation was assessed in vivo using Carrageenan-induced paw edema according to Lu et al. [35]. Male Swiss Albino mice (25–30 g) were randomly grouped into six groups (*n* = 6). The different groups were pre-treated orally as follows: Group 1 served as a negative control (left paw) and carrageenan-induced paw edema model (right paw), which received isotonic saline solution at 10 mL/kg body weight (bw), and Group 2 was given the non-steroidal anti-inflammatory drug Indomethacin (10 mg/kg, bw) and considered as a positive control. Tested groups 3, 4, 5 and 6 were treated with the respective doses of 200 and 400 mg/kg (bw) of each studied extract. After 30 min of the pre-treatment, the paw edema was induced in the right hind paw of each animal of all groups by a sub plantar injection of 0.1 mL of carrageenan suspension (1%), freshly prepared in normal saline solution.

#### 2.7.3. Estimation of Inhibition Edema Rate

The diameter of the edema was calculated using a digital caliper before and after 1 h, 2 h, 3 h, 4 h and 5 h of the carrageenan administration. The following equation (Equation (4)) was used to calculate the edema’s inhibition rate:

I (%) = [(MEE_control_ − MEE_treat_)/MEE_control_] × 100. (4)

MPE_control_: mean paw edema volume in the carrageenan-induced edema control group at a given time; MPE_treat_: mean paw edema volume in the tested group at a given time.

### 2.8. Ethanol-Induced Gastric Mucosal Damage

The anti-ulcerogenic activity of RAE and RMA was evaluated using a EtOH-induced gastric ulcer model on male Swiss Albino mice (25–30 g), as explained by Li et al. [36]. Mice were randomly divided into six experimental groups (*n* = 6) and deprived of food for 12 h with free access to water before dosing. At the beginning of the experiment, animals were dosed as follows: Group 1 and 2 received normal saline solution at a dose of 10 mL/kg (p.o.) and served as negative control and an EtOH-induced gastric ulcer model, respectively. Group 3 served as a positive control and received Omeprazole (20 mg/kg, p.o.), and Group 4, 5, 6 and 7 received the respective doses of 200 and 400 mg/kg (p.o.) of each studied extract. One hour after pre-treatment, the ulcer was induced by a single oral administration of EtOH (90%) at 10 mL/kg.

#### 2.8.1. Estimation of Ulcer Index (UI)

One hour after EtOH-ulcer induction, animals were sacrificed and their stomachs were removed and washed immediately with normal saline solution (NaCl 0.9%). The excised stomachs were opened and examined macroscopically for the ulcer index (UI). The length of each ulcer lesion was measured (mm) and the degree of gastric mucosal damage was investigated by scoring the number and severity of gastric ulcer lesions as follows: (0) no ulcer (0 mm); (1) 1–3 smell lesions (<10 mm); (2) 1–3 large lesions (≥10 mm); (3) 1–3 thickened lesions; (4) more than 3 smell lesions; (5) more than 3 large lesions. The ulcer index of each group was determined as the total of the sum of the scores and divided by the number of animals. In addition, the preventive percentage (PP %) of the pretreatments was calculated using the following equation (Equation (5)):

PP % = [(UI_control_ − UI_treated_)/UI_control_] × 100 (5)

#### 2.8.2. Histopathological Examination

At the end of the experiment, animals were sacrificed using cervical dislocation and the right paw/stomach of each animal from each group was removed and washed with ice-cold saline and fixed in buffered neutral formalin 10%. Tissue sections of 5 µm were mounted on glass slides using standard techniques and stained with hematoxylin and eosin 2% for an examination microscopically under a light microscope equipped for photography.

### 2.9. Statistical Analysis

All in vitro experiments were performed in triplicate and the results were expressed as mean ± SD. The in vivo experiments were carried out in groups of six animals, except the acute toxicity, and the results were expressed as mean ± SEM. Analysis of variance was performed by one way ANOVA followed by Tukey’s multiple comparison tests, using Graph Pad Prism version 8.0.2 (Graph Pad software Inc, San Diego, CA, USA). The statistical significance was determined at *p* < 0.05.

## 3. Results

### 3.1. Spectrophotometric Determination of Total Phenolic Content

Aqueous and methanolic crude extracts of *R. tuberculata* were subjected to determining their total phenolic content. Regarding the obtained results, both studied extracts contained diverse classes of bioactive secondary metabolites but with different amounts. Generally, RME extract revealed significantly (*p* < 0.05) the highest levels of TPC (53.4 ± 0.01 mg GAE/g d.E), TFC (8.1 ± 0.02 mg QE/g d.E) and CTC (30.7 ± 0.4 mg CE/g d.E) than that recorded by RAE. Overall, this last extract seems to be the poorest in term of most secondary metabolites’ classes, with TPC, TFC and CTC values of 46.9 ± 0.04 mg GAE/g d.E, 6.2 ± 0.07 mg QE/g d.E and 22.3 ± 0.3 mg CE/g d.E.

### 3.2. cLC-DAD Quantitative Analysis

**Figure 1:**
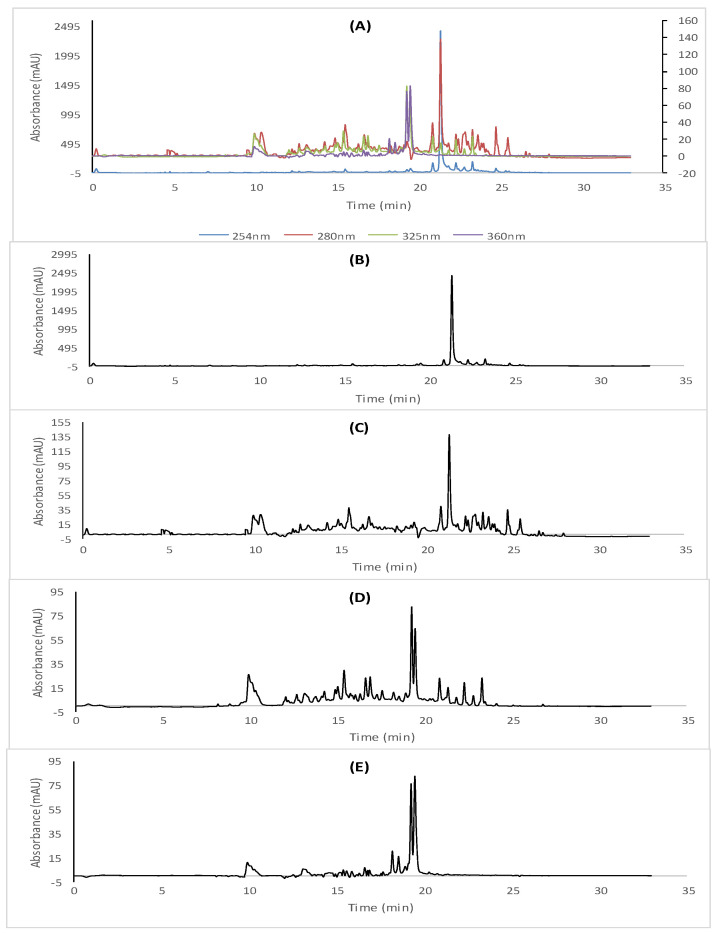
LC−DAD chromatogram of secondary metabolites from *R. tuberculata* aqueous extract. (**A**) Global chromatograms detected at four wavelengths: (**B**) at 254 nm, (**C**) at 280 nm (**D**), at 325 nm and (**E**) at 360 nm.

**Figure 2:**
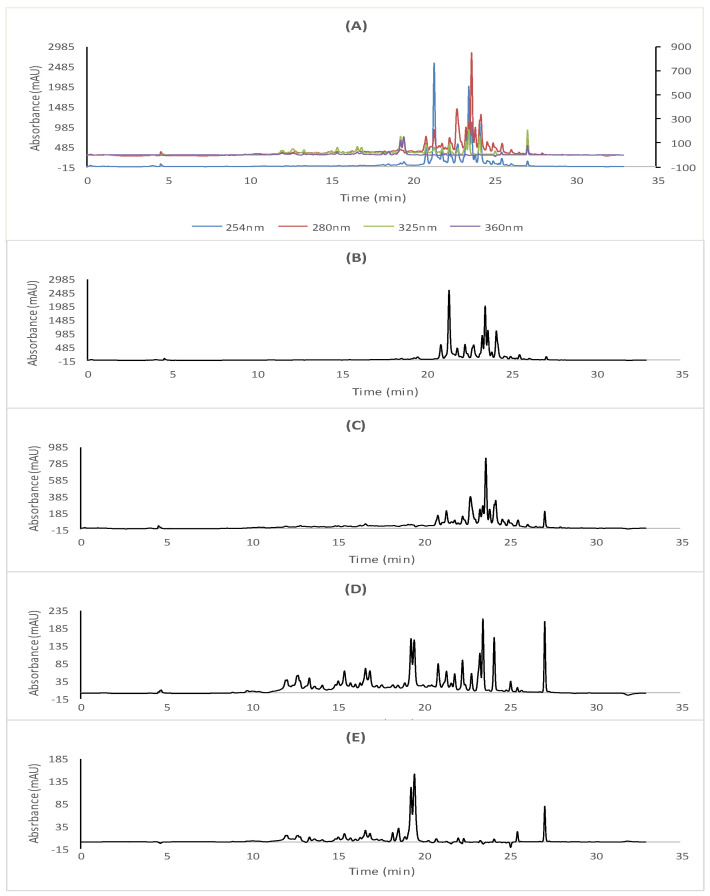
LC−DAD chromatogram of secondary metabolites from *R. tuberculata* methanolic extract. (**A**) Global chromatograms detected at four wavelengths (**B**) at 254 nm, (**C**), at 280 nm (**D**), at 325 nm and (**E**) at 360 nm.

In the present study, *R. tuberculata* polar extracts were submitted for the first time to cLC-DAD quantitative analysis, and the obtained cLC-DAD chromatograms of RAE and RME have been illustrated in Figure 1 and Figure 2, respectively. Based on molecular separation at four wavelengths, UV-spectra and retention time, both *R. tubercutata* aerial parts’ extracts seemed to be rich in many classes of polyphenolic compounds, as summarized in Table 1. The most abundant phenolic acids were found in RAE extract, which were the gallic acid (264.16 mg/100 g d.E) as the major phenolic acid in this extract followed by trans-cinnamic acid (112.07 mg/100 g d.E), cinnamic acid (25.38 mg/100 g d.E) and *p*-hydroxybenzoic acid (21.32 mg/100 g d.E). Moreover, the vanillic and trans-ferulic acids were obtained in RAE at equal amounts (>10 mg/100 g d.E), in the presence of some traces of caffeic and *p*-coumaric acids. Likewise, it was noted that the highest amount of the trans-cinnamic acid (131.87) was detected in the RME, followed by cinnamic acid (100.99 mg/100 g d.E) and caffeic acid (27.14 mg/100 g d.E), with considerable presence of the gallic acid (24.53 mg/100 g d.E) in this extract. Additionally, *p*-hydroxybenzoic and vanillic acids were found at equal amounts (≥10 mg/100 g d.E). Nevertheless, the *p*-coumaric and trans-ferulic acids were detected as traces. 

Relevantly, the myricetin was quantified as the most abundant flavonoid in RME and RAE, with the respective amount values of 5368.64 mg/100 g and 2624.82 mg/100 g d.E, whereas sylimarin was, considerably, the second major flavonoid compound (>700 mg/100 g d.E) in both *R. tuberculata* extracts. However, RME was the richest extract in naringenin with the highest amounts of 69.97 mg/100 g d.E, followed by rutin (58.31 mg/100 g and 25.86 mg/100 g d.E). Indeed, quercetin and catechin were both detected in concerning extracts, in which the higher values were depicted in the RME (Table 1).

### 3.3. In Vitro Antioxidant Activity

In vitro antioxidant activity of the samples was practically linked to the assay models being achieved. In this study, six different methods were performed to investigate the antioxidant potential of RAE and RME from Algerian *R. tuberculata* aerial parts, based on different mechanisms of action such as ABTS radical cation and DPPH scavenging activity, ferric and cupric reducing power (FRAP, CUPRC), β-carotene-linoleic acid blanching inhibition and total antioxidant capacity (TAC). The obtained results were expressed as mean ± SD and summarized in Table 2 as ascorbic acid equivalents, IC_50_ and A_0.5_ values.

#### 3.3.1. Radicals Scavenging Activity

The obtained data showed that *R. tuberculata* aqueous and methanolic extracts exhibited remarkable radical scavenging activities, which were evolved in a dose-dependent manner against both DPPH and ABTS radicals. Interestingly, RME presents a better free radical DPPH and ABTS scavenger agent (*p* < 0.001), with the respective IC_50_ values of 53.78 ± 0.5 and 80.72 ± 0.9 μg/mL, than that exerted by the RAE (74.87 ± 1.1 and 143.54 ± 1.0 μg/mL, respectively), as appointed in Table 2. In fact, its DPPH antiradical activity was higher than that found by the BHA and ascorbic acid, but it remains statistically similar to that exhibited by the synthetic antioxidant (BHT) and the natural pure compounds (gallic acid and quercetin), used as a positive control. However, RAE revealed a moderate scavenging activity which was significantly (*p* < 0.001) weaker than that expressed by all the employed reference drugs. It is important to underline that among all these standards, the BHA significantly exhibited (*p* < 0.001) an excellent antiradical activity with the lowest IC_50_ value (15.74 ± 0.5 μg/mL) followed by ascorbic acid (26.38 ± 0.5 μg/mL), with no significant difference between them, while BHT, gallic acid and quercetin statistically present a similar activity against DPPH radical with respective IC_50_ values ranging between 49.77 ± 0.1, 53.03 ± 0.0 and 60.77 ± 0.0 μg/mL. In contrast to DPPH assay, BHT provided the greatest antioxidant activity against ABTS, with the lowest IC_50_ value of 1.55 ± 0.3 μg/mL, which is not significant compared to the ABTS scavenging effect of BHA (7.54 ± 0.7 μg/mL).

#### 3.3.2. Reducing Power

In both reducing power assays, *R. tuberculata* polar extracts revealed an effective reduction power on iron and copper ions, which was progressed in a concentration-depending manner. Based on the statistical findings (Table 2), RME and RAE similarly reduced the iron ions with A_0.5_ values reaching to 132.71 ± 1.1 μg/mL and 132.92 ± 0.9 μg/mL, respectively. In both studied extracts, they showed, significantly (*p* < 0.001), a moderate iron reducing power, with high FRAP values, when compared to the reference drugs used, which are ordered from the most to least excellent reducer agents, as follows: gallic acid > BHA > Ascorbic acid > quercetin > BHT. However, the greatest copper reducing power was registered by RAE (A_0.5_ = 196.91 ± 0.8 μg/mL), in spite of its moderate phenolic contents, compared to RME. Relevantly, synthetic antioxidants (BHA and BHT), as a positive control, displayed higher Cu^+2^ reduction activity (*p* < 0.001) than the concerning extracts, with the lowest A_0.5_ values of 3.64 ± 0.2 and 9.62 ± 0.9 μg/mL, respectively, with no significant difference (*p >* 0.05) between them.

#### 3.3.3. Lipid Peroxidation Inhibition Activity

In the present study, the β-carotene blanching inhibition activity of *R. tuberculata* extracts was investigated, where the BHA and BHT were used as antioxidant standards. Our findings revealed that both aqueous and methanolic extracts effectively inhibited the linoleic acid peroxidation phenomenon in a concentration-dependent manner. Apparently, RME seemed to possess a significant β-carotene blanching inhibitory effect with an IC_50_ value of 153.18 ± 0.06 μg/mL, which was two times lower than that recorded by RAE (254.58 ± 1.7 μg/mL). However, this inhibition activity of the β-carotene blanching remains lower than that exhibited by BHT and BHA, with an equal IC_50_ value (1.24 ± 0.0 and 1.26 ± 0.0 μg/mL), as reported in Table 2.

#### 3.3.4. Total Antioxidant Activity

Remarkably, RME seemed to possess the minimal TAC value (135.8 ± 0.02 AAE mg/g E), which is two times weaker than that expressed by RAE (369.57 ± 1.0 AAE mg/g E). Indeed, this significant TAC rate (*p* < 0.001) of RME, which is the highest in term of polyphenols, reflects its great antioxidant capacities compared to RAE, as summarized in Table 2.

### 3.4. Anti-Alzheimer Activity

The in vitro anti-Alzheimer activity of *R. tuberculata* aqueous and methanolic extracts was evaluated by estimating their inhibitory capacities against acetyl-cholinestrerase enzyme, and the Galanthamine, a natural AChE inhibitor frequently incorporated in therapeutics, was used as reference. The results were appointed as IC_50_ values ± SD and summarized in Table 3. In terms of concentration-dependent inhibition, RAE and RME markedly exhibited a significantly anti-AChE activity (*p* < 0.001) compared with a negative control, with maximum inhibition rate values ranging from to 89.7 ± 1.5 % and 82.3 ± 1.4 %, respectively, at the highest tested concentration (200 μg/mL). Statistically, RAE seemed to be (*p* < 0.01) an effective anti-AChE agent with the lowest IC_50_ value of 51.08 ± 1.6 μg/mL compared to RAE (56.68 ± 0.97 μg/mL). However, the neuro-protector activity of RAE remains lower than that recorded by Galanthamine (6.27 ± 1.1 μg/mL).

### 3.5. Acute Oral Toxicity

During the first 24 h of the toxicological study, it was observed that RAE and RME caused some behavioral changes, mostly the length of sleeping time with light anorexia, compared to the control group. Indeed, most of these visual signs were disappeared evidence for the remaining period of study. However, no mortality was noted in all treated groups, except once in the RME (5000 mg/kg)-treated group. Thus, the LD_50_ may be considered to be higher than the limit dose of 5000 mg/kg for both *R. tuberculata* extracts. Based on these results, *R. tuberculata* extracts are therefore classified in the Global Harmonization System for Chemical substances as few toxic drugs from category 5.

### 3.6. In Vitro and In Vivo Anti-Inflammatory Activity

#### 3.6.1. Bovine Albumin Denaturation Inhibition

**Figure 3:**
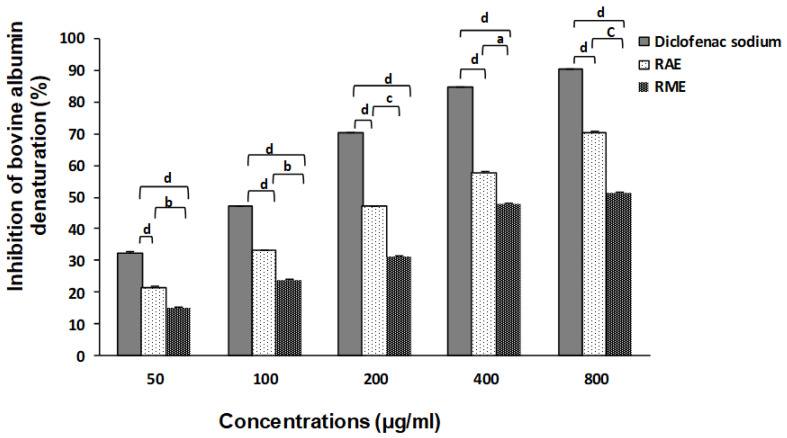
Effect of *R. tuberculata* extracts on albumin denaturation. Values were expressed as mean ± SD (*n* = 5). Mean values followed by different letters are significantly different (based on one-way ANOVA followed by Tukey’s multiple comparison tests. Level of Significance: ^a^ *p* < 0.05, ^b^ *p* < 0.01, ^c^ *p* < 0.001, ^d^ *p* < 0.0001).

The in vitro anti-arthritic activity of RAE and RME were assessed using bovine albumin denaturation model. Our findings showed that in both studied extract inhibited thermally-albumin denaturation whereas, the RAE has a remarkable preventive effect on albumin denaturation for all tested concentrations (*p* < 0.001), when compared to negative control. In fact, this anti-inflammatory effect was evolved in a dose-dependent manner and reaches its maximal inhibition rate value of 70.8 for RAE and 51.58% for RME, at the highest tested concentration (Figure 3). Relevantly, RAE presented an IC_50_ value of 212.27 ± 0.04 μg/mL, which was two time higher than that recorded by Diclofenac sodium (115.76 ± 0.19 μg/mL), an anti-inflammatory drug frequently used against arthritic diseases. However, the lower anti-arthritic activity was exhibited by RME, when compared to positive control, with an IC_50_ value > 400 μg/mL, as shown in Table 3.

#### 3.6.2. Carrageenan-Induced Paw Edema

The aqueous and methanolic extracts of *R. tuberculata* crude extracts exhibited significantly (*p* < 0.001) a dose-dependent suppression of edema induced by carrageenan, when compared to paw edema model group which was only treated with normal saline (Table 4) Statistically, the pre-treatment with RME inhibited effectively the evolution of paw edema on mice than RAE, especially during the second and the third hours following carrageenan injection. However, similar inhibition rates of edema were significantly produced by RAE and RME at the fourth hour, which were respectively 51.32% and 51.51% for the highest tested concentration. At the end of the experiment, RAE exhibited significantly a close edema inhibition rate (60.32%) to that presented by indomethacin, which revealed a maximum rate value of 60.37%. Moreover, the excellent capacity to suppress the paw edema phenomena (61.78%) was also noted for RME at a dose of 400 mg/kg which is slightly higher than that recorded by indomethacin. Noting that these results were considered highly significant (*p* < 0.001) when compared to carrageenan-induced paw edema model group.

### 3.7. Antiulcer Activity

**Figure 4:**
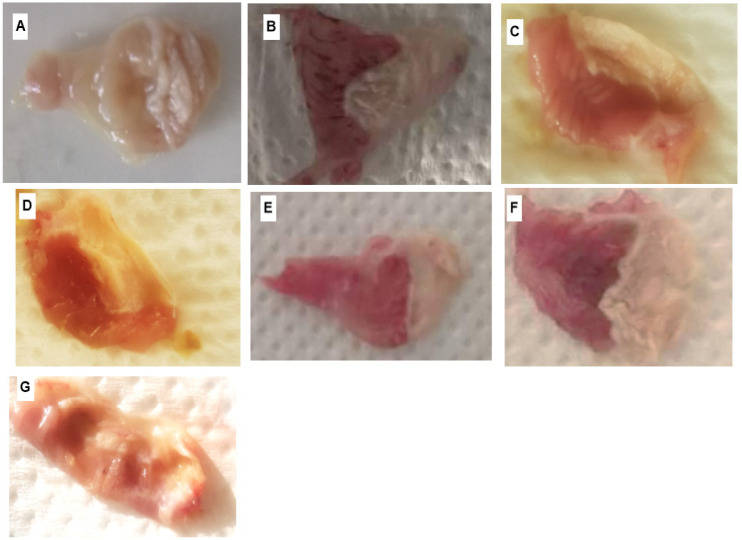
Macroscopic morphology of mice stomachs in ethanol-induced gastric damage model (H&E stain × 100). (**A**) Normal control (**B**) ethanol-control, (**C**) omeprazole (20 mg/kg), (**D**) RAE (200 mg/kg), (**E**) RAE (400 mg/kg), (**F**) RME (200 mg/kg), (**G**) RME (400 mg/kg).

The oral administration of ethanol at a dose of 10 mL/kg seemed to produce several ulcer lesions in the control mice stomachs as shown in Figure 4. Indeed, it was significantly related to an extensive ulcer index value (4.7 ± 0.24), compared to all tested groups (Table 5). However, the pre-treatment of animals with *R. tuberculata* extracts exhibited significant decrease in UI value (*p* < 0.001) in a concentration dependent manner. RAE at a dose of 400 mg/kg (*p*.o.) showed to have the best gastro-protective effect against ethanol-induced gastric mucosa lesions than RME (76.6 ± 1.0%) and presented the high ulceration inhibition rate value of 81.3 ± 0.6% which is statistically similar to that recorded by omeprezole (80.9 ± 0.7%).

### 3.8. Histological Assessment of In Vivo Anti-Inflammatory and Antiulcer Effects

**Figure 5:**
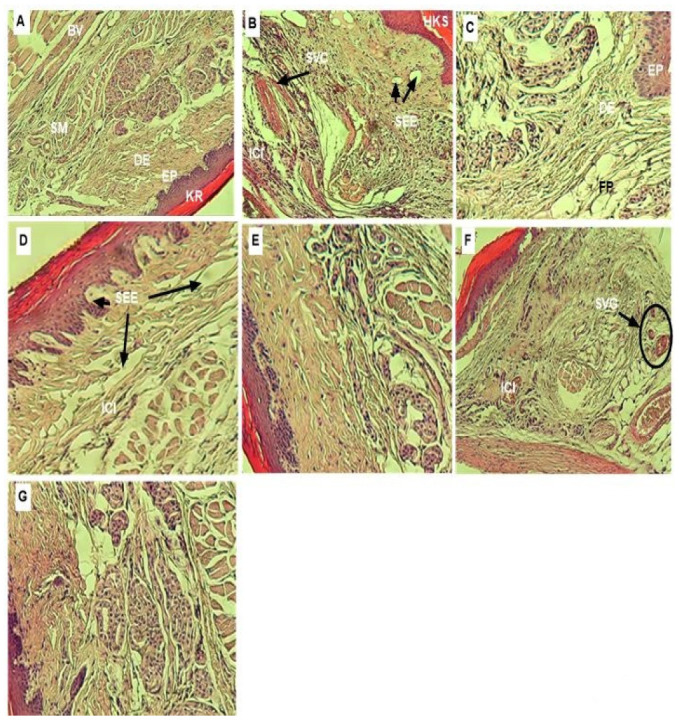
Histological examination of mice paws tissue in carrageenan-induced paw edema model (H&E stain, Magnification × 100).KR: Keratin. EP: epidermal layer. DE: dermal layer. FP: fatty part. SM: Striated muscle. (**A**) Normal control shows normal keratin (KR), normal epidermal and sub epidermal architecture. (**B**) Carrageenan-induced paw edema group shows massive inflammatory cells infiltration (ICI), hyper keratotic skin HKS, Sub epidermal edema (SEE) and several vascular congestions. Pre-treatment with Indomethacin (**C**) or (**D**) RAE and RME at dose of 400 mg/kg (**E**,**G**) prevent significantly the carrageenan-induced paw edema with no signs of acute skin inflammation. Treated groups with RAE (**E**) and RME (**F**) at 200 mg/kg show few cell infiltration and minor vascular congestions which suggest onset of inflammatory response phenomena.

The histopathological examination of paw tissues using H&E staining revealed that the left paws of the animals from group 1, which were non-inflamed and considered as negative control, were characterized by normal morphology, kiratinized skin, intact striated muscle tissue and bone trabeculae conveniently placed around of medullar spaces, without appearance of edema in upper part of foots (Figure 5A). As opposed to that, epidermal hyperkeratosis, several congestions of blood vessels, massive neutrophils and plasma infiltration and edema formation were observed in the left foots during the five hours after mice immunization with carrageenan (group 1), which reflects the strongly swollen of tissue and the suffering of cells before their necrosis at the base of the inflamed foot pads (Figure 5B). However, the pre-treatment with the indomethacin (20 mg/kg, p.o.) markedly alleviated carrageenan-induced paw thickness and improved epidermal edema (Figure 5C). The same preventive effect was observed in paw tissue of RAE (200 and 400 mg/kg) and RME (400 mg/kg, p.o.) treated groups (Figure 5D,E,G), respectively, which ameliorated the edema tissue. While, mild vascular alterations authoring the blood vessels and slight cell infiltration were observed in limited areas for mice of RME (200 mg/kg)-treated group; evertheless, the tissue integrity was generally preserved (Figure 5F).

**Figure 6:**
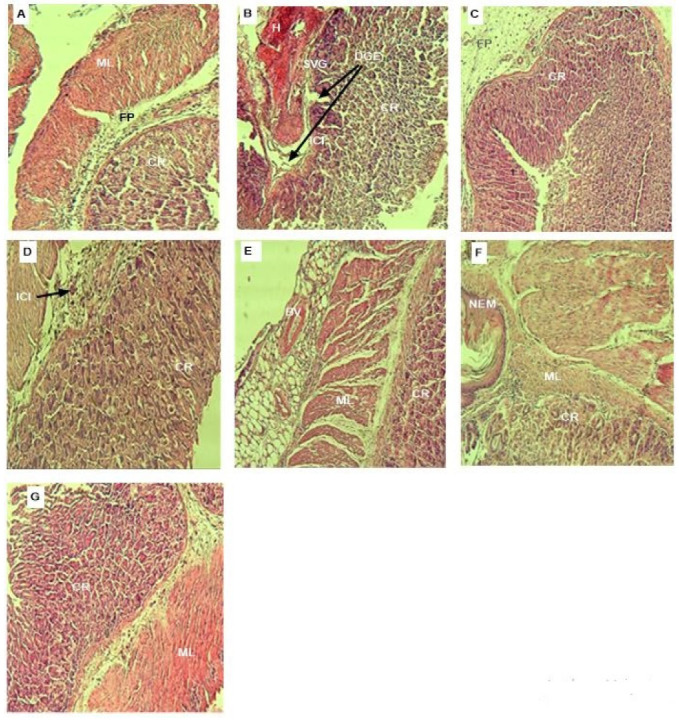
Histological examination of mice stomach tissues in Ethanol-induced gastric damage model (H&E stain × 100). (**A**) Normal control shows normal morphology of mucosal epithelium (**B**) ethanol-control, (**C**) omeprazole (20 mg/kg), (**D**) RAE extract (200 mg/kg), (**E**) RAE extract (400 mg/kg), (**F**) RME extract (200 mg/kg), (**G**) RME extract (400 mg/kg). CR: corpus. FP: fatty part. ML: muscle layer.

Concerning the anti-ulcerogenic activity, the histopathological analysis (H&E stain × 100) showed that no signs of submucosal edema formation or epithelial cell loss were observed in the stomachs of mice in normal control group (Figure 6A). In contrast, high degree of epithelial cell damage as well as several congestions and haemorrhagic path production were found in ethanol- induced gastric ulcers model group (Figure 6B). However, the pre-treatment of mice with Omeprazole (20 mg/kg) as standard (Figure 6C) or both RAE and RME (Figure 6E–G), respectively, preserved the corpus architecture and muscle layer of the stomachs in ulcerated animals, which improved inflammatory response and inflammatory cell infiltration to the sub-mucosal layer. Indeed, both studied extracts could alleviate the ethanol-induced stomach damage in a dose dependent-manner, and prevented effectively the gastric epithelial destruction especially at the highest tested dose of 400 mg/kg p.o.

## 4. Discussion

Currently, several pharmacological investigations suggest the beneficial proprieties of medicinal plants, which were traditionally used as folk medicine to treat several types of chronic disease, especially polyphenols. Interestingly, it has been reported that these natural biocompounds possess a potent capacity to prevent or alleviate the acute and chronic disorders related to the massive production of reactive oxygen species, as well as free radicals. Indeed, it has been affirmed that free radicals can cause many inflammatory diseases, such as rheumatoid arthritis, gastric ulcers and Alzheimer’s disease, or participate in their development [20]. However, the use of synthetic antioxidants and non-steroidal anti-inflammatory drugs is undesirably perceived by consumers, and the main reason is their possible carcinogenic and toxic effects [5,37].

Several phytochemical studies reported on the richness of *Ruta* species in polyphenols and flavonoids [3,13,17,18], while a limited number of reports on phenolic screening of *Ruta tuberculata* (or *Hapophyllum tuberculatum*) could be found in scientific literature. In this context, the actual lack of studies that focus on the phenolic characterization in different extracts of *R. tuberculata* from Algeria increased our interest and curiosity to study it in depth and motivated us to characterize the phenolic compounds of crude polar extracts of their aerial parts, using cLC-DAD quantitative analysis, but also to evaluate their pharmacological effects using both in vitro and in vivo approaches.

In agreement with our findings, Zengin et al. [38] reported that the water extraction of *Hapophyllum myrtifolium* (Rutaceae) aerial parts by decoction method provided the highest extraction yield (38.47%), while those obtained by methanolic maceration was only (18.83%). However, their finding values were higher than those actually obtained in our study. In contrast, Kacem et al. [39] confirmed that the ratio of methanol:water (1:1) is the best system solvent used to obtain the maximum extraction yield (14.5%) from Tunisian *R. chalepensis* aerial parts, which was slightly higher than our results. On the other hand, their investigation via water-based extraction via the infusion approach provided only a yield value of 10.36%, which is lower than our findings.

Depending on the polarity of the solvent systems used in this study, the studied extracts revealed differences in total phenolic contents, flavonoids and tannin. Indeed, our results were in accordance with those documented by Hamdi et al. [18] on the aerial parts of Tunisian *R. tuberculata*, who demonstrated that the highest TPC and TfC amounts were found in its MeOH (100%) extract (126 GAE/g d.E and 36.6 QE/g d.E) when comparing to the aqueous extract (117 GAE/g d.E and 16.6 QE/g d.E), hence, they were higher than that obtained in our study. Otherwise, Eissa et al. [17] indicated that the TPC found in EtOH (70%) extract of Egyptian *R. tuberculata* aerial parts was 46.2 mg GAE/g d.E which is lower than those recorded for both targeted extracts. According to our results, the respective TPC (120.5 and 142.5 mg GAE/g d.E) and TFC (4.01 and 9.32 CEs/g d.E) amounts investigated by Kacem et al. [39] in aqueous and MeOH:H_2_O (1:1) extracts of Tunisian *R. chalepensis* aerial parts were higher than those recorded by the target extracts, whereas, the respective CTC amounts (14.69 and 13.89 CEs/g d.E) for these extracts were weaker than those obtained in the current study. In addition, Zengin et al. [38] found that both aqueous and methanolic extracts of *Haplophyllum myrtifolium* have nearly similar TPC (34.5 and 39.2 GAE/g d.E) amounts, which were also weaker than our findings. In addition, the report data of another researcher [2] showed that the aqueous extract of Algerian *R. chalepensis* was the lowest in total phenolic (12.2 GAE/g d.E) and flavonoid contents (3.43 mg QE/g d.E) as compared to other studied fractions. When referring to these findings, the aqueous extract of *R. tuberculata* seemed to be richest than this one.

Overwhelming evidence has demonstrated that many factors, involving the genetic inheritance, extraction method and the solvent system concept, may affect the extraction yield and influence the diversity and nature of chemical contents between *Ruta* plants [12,24,40]. Indeed, the chemical proprieties of different secondary metabolite classes significantly influence their solubility and consequently their extractability yields [7]. From these results, we can suggest that hydro-alcoholic solvent was mostly the suitable system used to extract the maximum of recovery vegetal secondary metabolites with high and middle polarity, in particular, the diverse phenolic classes which are well known to possess various pharmacological proprieties such as antioxidant, anti-inflammatory, antiulcerogenic and neuro-protective activities.

In this context, one of the objectives of the present study is to unequivocally corroborate the amount of phenolic components of RAE and RME beforehand identified by Saidi et al. [3] using LC-MS/MS system, since the quantification of all targeted components using LC-MS/MS was impossible, especially for those present at weak concentrations and, thus, below the quantification limits of this method. We further analyzed these extracts using cLC-DAD method. Therefore, the combination of DAD and MS/MS detection systems allowed us to bring new compositional data about the contained polyphenols in *R. tuberculata* aerial parts. Actually, the most abundant small phenolic acids were found in RAE, in which the gallic acid was detected only by cLC-DAD system as the major phenolic acid in this extract followed by trans-cinnamic acid, cinnamic aid and *p*-hydroxybenzoic acid, which could explain the high sensitivity of this method for the detection of these components. Likewise, it was noted that the highest amount of trans-cinnamic acid was detected in the RME, followed by cinnamic acid and caffeic acid with considerate presence of the gallic acid in this extract. Based on the evidence findings, the diversity of flavonoids classes was also observed in both studied extracts. Interestingly, RME seemed to be the richest extract in naringenin, rutin and myricetin, thus, this letter was quantified as the major flavonoid compound in both targeted extracts, followed by sylimarin. Moreover, quercetin and catechin were only determined by cLC-DAD in both concerning extracts, in which the higher amounts were quantified in RME. Indeed, the richness of this extract in flavonoids content has been confirmed by both analysis methods [3].

According to our phenolic screening results of RME, the same varieties of phenolic compounds were also identified by Eissa et al. [17] in Egyptian *R. tuberculata* EtOH (70%) extract using HPLC-MS, which were described the presence of the derivatives of the benzoic acid and cinnamic acid. Moreover, many flavonoid classes, mainly flavones derivatives such as 3-hydroxymethoxyflavone, methoxy-luteolin glucoside and methoxy-apigenin glucoside, and flavonols derivatives, in particular, syringetin and quercetin glucuronide were also identified in this extract [17]. In addition, our finding results effectively align with those previously documented on EtOH (80%) extract of *R. graveolens* L. which was subjected to LC-MS/MS analysis, noting that two flavonoid compounds were identified, namely rutin and quercetine [12]. Using the same technique analysis, hesperidin and rutin were detected as main abundant flavonoid compounds followed by rhamnitin and quercetin in EtOH extract of *R. chalepensis* leaves from northern Tunisia [41]. Whereas, RP-HPLC analysis of the MeOH extract of cultivated and spontaneous *R. chalepensis* monitored the presence of 12 different phenolic acids and 7 flavonoids [42]. This information could support completely our suggestion about the presence of great diversity of molecules, with chemical structure of phenol, that were identified in *R. tuberculata* aerial parts but possibly with differences in metabolite amounts when compared to the others *Ruta* species as reported in previous studies. In fact, the chemical composition of secondary metabolites and their contents in *Ruta* plants can vary under several factors such as genetic inheritance, location and semi-arid climatic conditions, the developmental stages of the plants, harvest season, used solvents and extraction method [12,18,24,43].

No universal methods could be able to evaluate particularly the antioxidant activities of plant extracts. In current study, six spectrophotometric bioassays were carried out to estimate the possible antioxidant capacities of *R. tuberculata* crude extracts, that would be more informative but not in a precise manner. Practically, the high scavenging effect of alcoholic and hydro-alcoholic extracts of *Ruta* species, especially *R. tuberculata* was reported by previous studies using different approaches [1,17,18,41,42]. Compared to their findings, we suggested that the promising antiradical capacity of *R. tuberculata* extracts could be attributed to the presence of various bioactive phenolic metabolites. Indeed, the obtained results corroborate the correlation between free radical scavenging effect and the phenolic content in *R. tuberculata* extracts in particular cinnamic, trans-cinnamic and gallic acids. This latter is a small phenolic compound mostly abundant in RAE, known for its strong antioxidant effects [44]. In addition, it has been reported that hydrogen or hydroxyl groups containing phenolic compounds are able to easily donate the acidic protons to stabilize free radicals, while the delocalization of unpaired electrons leads to the formation of a stable phenoxyl radical [24,45]. Moreover, the substantial free radical scavenging effect of *R. tuberculata* extracts could related also to their identified flavonoid compounds. Previous studies afforded further evidence that the presence of double bound between C2 and C3 in C ring and hydroxyl groups in C3 and C4 in B ring of flavonoid compounds structure are important features for high antiradical effect in flavonoids [24,46].

There are no data available in the literature about the reducing power property of *R. tuberculata* extracts. To the best of the authors’ knowledge, this is the first time that the reducing power capacity of *R. tuberculata* extracts was determined using FRAP and CUPRAC assays. Based on our results, both tested extracts exhibited an excellent reducing power effect compared to FRAP values (≥900 μg/mL) reported by Ouerghemmi et al. [42] on MeOH extracts of leaves and flowers of cultivated and wild *R. chalepensis* from Tunisia. Furthermore, our findings showed that both studied extracts potently reduced the iron ions compared to EtOH extract of Algerian *R. chalepensis*, which revealed a moderate FRAP activity (FRAP value of 140 μg/mL). Whereas, it was demonstrated that its aqueous extract has been inactive on both iron and copper, reducing power bioassays with an A_0.5_ values > 200 μg/mL [2]. In fact, the reducing power capacities that possess *R. tuberculata* extracts are probably linked to their polyphenols content, which act similarly as redactors via their electron donating ability. Relevantly, it has been documented that the main antioxidant compounds responsible for the reducing power of plant extracts are the small phenols, based on their power to provide more atoms and, consequently, to effectively reduce the ferric and cupper ions [7,46]. Therefore, it could be stated that the antioxidant potential of polyphenols is strongly related to their redox proprieties, where they act as free radical scavengers through hydrogen and electron transfer mechanisms, as metal ions’ binding agents and as modulators of the endogenous antioxidant systems [45].

In β-carotene assay, the auto-oxidation of linoleic acid in the presence of oxygen leads to the formation of lipid free radicals, which oxidases and, consequently, decolorizes the β-carotene. However, the addition of an antioxidant substance may scavenge these radicals and delay the β-carotene depolarization process [7,46]. In the present work, we found that RME exhibited an excellent β-carotene blanching inhibitory effect, but remains lower than that recorded with BHA BHT, as antioxidant standards. Indeed, many phyto-pharmacological data [2,18,39] documented that aqueous and alcoholic extracts of some *Ruta* species recorded higher IC_50_ values than those found in this study, which reflects evidence of the high inhibition effect of RAE and RME on lipid peroxidation.

Indeed, we noticed that the antioxidant capacity of *R. tuberculata* extracts was remarkably correlated with their bioactive phenolic compounds, which could serve as hydrogen-transferring donors. Our suggestion is in accordance with previous data [7,13,18]. Indeed, these reducers are able to react with lipid radicals, as well as certain precursor peroxides, to convert them into more stable products that could terminate the free radical chain reaction and prevent lipid peroxidation [39]. In addition, previous reports monitored that less polar and nonpolar fractions have a more interesting β-carotene blanching inhibition rate than aqueous extract. Hence, it is mostly dependent on the type of solvent used during extraction, which can be explained by the presence of lipophilic bioactive compounds that are able to neutralize free radicals in this test [2,39]. Actually, β-carotene reaction medium is a linoleic acid emulsion that can allow the nonpolar bioactive molecules, such as triterpenoids, to access freely lipid radicals [7,47]. It was documented also that alcoholic solvents are able to dissolve some diterpenes and flavonoid aglycones [7,47].

For the TAC assay, our findings showed that RME exhibited the best TAC rate, which presented the lowest TAC value compared to RAE. Unlike our results, previous data [2] assumed that the aqueous extract of *R. chalepensis* showed the weaker TAC (54.55 AAE mg/g E) than EtOH extract, whereas this letter has a closer TAC value to actual studied RME. Furthermore, Kacem et al. [39] affirmed that the TAC proportion was found to decrease in this order: EtOH > MeOH (50%) > MeOH (100%) > EtOAc > water > n-hexane extracts. Therefore, the variation in antioxidant capacity between studied extracts may strongly correlate with the higher amount of the same flavonoid compounds in RME than in RAE, which may act as reducer agents.

A study made by Zerin et al. [48] on alveolar A549 cells revealed that quercetin may possess the capacity to reduce oxidative damage induced by paraquat by regulating the expression of key antioxidant genes, especially the activation of transcription factor Nrf2 and its target HO-1 expression. These researchers noted an important decrease in ROS level and a significant increase in GSH level. A recent study also reported that quercetin may exert a noticeable protection against lipopolysaccharide-induced intestinal oxidative stress in broiler chickens through the activation of the Nrf2 signaling pathway, which, in return, had considerably downregulated the plasmatic levels of 8-hydroxy-2′-deoxyguanosine (8-OHdG), reactive oxygen species (ROS) and malondialdehyde (MDA) [49].

Catechin and epicatechin also play a crucial role in the prevention of mitochondrial oxidative stress induced by amiodarone, and this was proved using human lung fibroblasts cells (MRC-5) [50]. These researchers also noted that these two phytocompounds may also restore the activity of mitochondrial complex I and ATP biosynthesis by regulating the imbalance in superoxide dismutase and catalase activities. This information is very important, since damaged mitochondria produce ROS, especially in the form of superoxide anion (O2^−^) and hydrogen peroxide (H_2_O_2_).

It is also interesting to underline that caffeic acid may prevent the pro-oxidant effects of a high iron dose and reduce hypercholesterolemia but also increase vitamin E levels in plasma, and this effect could be generated due to the high bioavailability of this polyphenol [51]. Another study made by Gutierrez-Zetina [52] reported the beneficial effect of this compound. 

Various factors such as temperature increase, pH level variation, exposition to heavy metal or to UV light and radiation can intensively alter the molecular structure of the proteins that will deprive their secondary and tertiary structure, thus, it leads to loss of their vital functions and often causes self-antigen generation in autoimmune dysfunction cases, such as rheumatic and inflammatory disorders [53,54,55]. For this purpose, the possible in vitro anti-arthritic potential of RAE and RME on thermally-induced albumin denaturation was investigated in this work.

Based on the results of the current study, we suggest that the preventive effect of *R. tuberculata* extracts, mainly of RAE, against thermally-induced albumin denaturation reflects its capacity to maintain the protein’s three-dimensional structure and, thus, to prevent proteins’ alteration, which would modulate even the generation of self-antigen. Indeed, RAE has been the effective inhibitor on albumin denaturation, which indicates that this extract could be one of the most interesting bio-resources of natural anti-arthritic agents. Therefore, this anti-inflammatory effect might be attributed to its content of actual bioactive compounds, such as polar flavonoids and small phenolic acids, and mainly gallic acid, which is known for its important antioxidant and anti-inflammatory effects [44]. Moreover, Mouffouk et al. and Sęczyk et al. [56,57] documented that these biocompounds can significantly slow down the proteins’ denaturation process by stabilizing their dynamic structure and protecting them from the oxidative stress generated by nitric oxide synthase (NOS) and massive ROS production, as well the chronic inflammatory responses such as autoimmune diseases, necrosis and certain arthritic disorders, which may accelerate the denaturation process of proteins.

The remarkable antioxidant potential and the significant in vitro anti-inflammatory activity, as well as the richness of RAE and RME in diverse phenolic compounds classes, motived us to investigate these extracts in depth on male mice using in vivo anti-inflammatory and anti-ulcerogenic activities on carrageenan-induced paw edema and EtOH-induced gastric ulcers models, respectively. However, the acute oral toxicity was previously performed in mice at low limit doses (2000 and 5000 mg/kg b.w (p.o.), in order to confirm the safety of these extracts by estimating their LD_50_ and possible therapeutic doses.

During the acute toxicity experiment, some treatment-related behavioral changes were observed in the RAE (2000 mg/kg)-treated group, and a single mortality was noted in the RME (5000 mg/kg)-treated group. Indeed, the presence of alkaloids in *R. tuberculata* extracts, especially in the aqueous extract, which may be the richest one of them [8,14], may explain the acute toxicity signs observed in these groups. According to the OECD guideline, the LD_50_ for both extracts is above the dose of 5000 mg/kg, which allow their use in complete safety at the selected therapeutic doses in the present study.

Indeed, when the extract was injected in the paw of an animal, carrageenan may induce biphasic acute skin inflammation, which contributes to the formation of edema. It is the most suitable model to evaluate the possible anti-phlogistic effect of administrated extracts and their content in biocompounds which have an anti-phospholipase A2 activity. Indeed, during the initial hour of the inflammatory process, many pro-inflammatory mediators were released, such as histamine and serotonin, to allow increase in vascular permeability, then, after that and up to the fourth hour, prostaglandins E2 (PGE2) and leukotriene would be produced by cyclooxygenases (COX-1 and COX-2) and 5-lipoxygenase (5-LOX), respectively, from the arachidonic acid, as a key product of phospholipase A2 [5,58]. This letter is the target of NSAIDs to interfere effectively in chronic inflammatory diseases. However, the use of these drugs over a long period of time may present some undesirable secondary effects [7].

In the current study, both *R. tuberculata* crude extracts exhibited a significant (*p* < 0.001) suppression of edema induced by carrageenan in a dose-dependent manner, which may explain the absence of any signs of inflammation in the histological sections corresponding to theses extracts compared to that of the carrageenan-control group. Moreover, this finding indicates that both extracts may contain some bioactive compounds able to inhibit edema formation, therefore, they may possess an anti-phospholipase A2. It is interesting to underline that the pre-treatment with RME more effectively inhibited the evolution of paw edema on mice than RAE, especially during the second and the third hours following carrageenan injection. Note that these findings were effectively correlated with the richness of RAE in flavonoid compounds, such as myrecitin, sylimarin, naringenin and rutin, which are known for their anti-inflammatory and antioxidant activities. Indeed, our findings were consistent with those reported by Ratheesh et al. [19], who demonstrated that MeOH extract of *R. graveolens* L. exhibited an excellent anti-edematous effect on adjuvant-induced arthritis in rats compared to the indomethacin group, considered a positive control. These researchers also reported that the oral administration of this extract can decrease edema evolution, cell influx and mediators release, but also can increase antioxidant enzymes’ activity and thus reduce free radicals’ production. Moreover, Sabry et al. [59] documented that the essential oil of *H. tuberculatum* growing in Libya has a great anti-inflammatory effect on carrageenan-induced paw edema in rats, in which a high percentage of edema inhibition was observed.

At the end of the experiment, RAE significantly exhibited a close edema inhibition rate to that exerted by indomethacin, while RME appears to be more effective than indomethacin. Therefore, this result reflects a potent anti-inflammatory effect, which has the RME on the late inflammatory stage and confirmed the presence of natural phospholipase A2 inhibitor compounds in this extract. The actual bioactive compounds included in plants, especially those with flavonoids, triterpenoid, and saponins, in nature can greatly interfere in the pathophysiology of inflammation and prevent the production of many pro-inflammatory mediators, such as cyclooxygenases (COX-1 and COX-2), 5-lipoxygenase (5-LOX), prostaglandins E2 (PGE2) and some key cytokine such as interleukin (IL)-1β, but also can downregulate the expression of mPGES-1, which could be effective to progressively replace non-steroidal anti-inflammatory drugs’ usage [19,58,59].

Gastric ulcers are the most common pathologies, that generally result from excessive alcohol consumption, ingestion of NSAIDs and severe stressful events (Chung et al. [60]). As far as the authors know, the present work is the first investigation which evaluates the possible gastro-protective effect of the polar crude extracts from Algerian *R. tubercultata* against acute ethanol-induced gastric ulcer lesions in mice. Our findings showed that both extracts exhibited, in a dose-dependent manner, a significant (*p* < 0.001) gastro-protective effect. Interestingly, RAE seemed to be more effective than RME, in despite of their low polyphenols content for all tested doses. Compared to the Omeprazole-treated group, the remarkable antiulcer effect was observed in the group which was treated with RAE (400 µg/kg, p.o.), with a high preventive rate, indicating that the pre-treatment with this extract may effectively prevent the formation of submucosal edema in mice, which reflects the high reduction in ulcer area on histological sections. Noting that the anti-ulcer effect of this extract was not correlated with its phenolic content, and this could be explained by the presence of other bioactive compounds such as hydro-soluble alkaloid, coumarin derivatives and small volatile essential oils, but also the hydrolysable tannins, which seem to possess non-negligible antioxidant and anti-inflammatory effects. According to our suggestion, Sabry et al. [59] demonstrated that mono-terpenes may act in vivo to reduce the normal inflammatory response, whereas terpinen-4-ol can modulate the vasodilatation and plasma infiltration induced by histamine, but may also slow down lipid peroxidation in order to prevent vascular endothelium damage. However, Ratheesh et al. [19] reported that alkaloids in *R. graveolens* L. exhibited an excellent anti-inflammatory effect via the reduction in edema evolution, cell influx and mediators release, but also by reinforcing antioxidant enzymes’ activities and decreasing free radicals’ generation. In addition, the hydrolysable tannins, such as gallic acid units esterified by sugar, seemed to be potent antioxidant, antibacterial and astringent agents, but these compounds may also possess the potential to bind with proteins on the somatic epithelial layer, which may explain their high antiulcer effect on the surface of gastric tissue, which contributes to decreased hyperacidity, excessive free radical accumulation and bacterial infections.

A study made by Mascaraque et al. [61] revealed that rutin may exert potent intestinal anti-inflammatory effects by downregulating the expression of key proinflammatory genes, including S100A8, TNFα, IFNγ, CXCL1 and IL-1β, but also by decreasing the activation of splenic CD4+ cells in order to control the activity of T lymphocyte. 

It was also reported that gallic acid can help in the treatment of rheumatoid arthritis by controlling the apoptosis process of fibroblast-like synoviocytes [62,63]. In addition, this phenolic acid can considerably reduce paw edema in a dose dependent manner but also limit inflammatory symptoms associated with ulcer by decreasing the levels of key pro-inflammatory cytokines, namely TNF-α, IL-1, IL-6 and IL-10, through modulation of NF-κB, MAPK and JAK-STAT signaling pathways [64]. Another study made by Tanaka et al. [65] on the histological level showed that gallic acid can suppress inflammation and adipocyte hypertrophy caused by the interaction between adipocytes and macrophages via a strong inhibition exerted by this compound on the expression of a key gene called monocyte chemoattractant protein-1 (MCP-1).

The anti-inflammatory activity of Kaempferol was also reported. Indeed, a study made by Liu et al. [66]. demonstrated that this flavonoid can considerably reduce the gene expression of a key pro-inflammatory cytokine called Eta-1 via the inhibition of the aldosterone signaling pathway, and this was proved using human umbilical vein endothelial cells (HUVECs). It is also interesting to note that a nutritional-based clinical trial made on diabetic patients reported that a kaempferol-rich bean diet can significantly reduce the plasmatic level of key inflammatory biomarkers such as C-reactive protein (CRP), IL-6 and TNF-α [67].

Synthetic and semi-synthetic acetyl-cholinesterase inhibitor agents (AChEIs) are clinically prescribed to patients who suffer from neurological disorders such as Alzheimer’s disease as a first step in the treatment process, but almost all of them showed some adverse secondary effects, mainly hepatotoxicity and gastric disturbances [1].

No reported data were found in the literature on the anti-AChE effect of *R. tuberculata* polar extracts. However, this neuro-protective activity of other species of the *Ruta* genus was demonstrated. In agreement with our findings, the aqueous extract of *R. montana* leaves from Tunisia revealed significant AChE inhibitory activity, which was more pronounced than the EtOH extract, with IC_50_ values of 57 ± 1.6 and 76 ± 1.6 μg/mL, respectively [1], despite their low phenolic content. Moreover, our results showed that both extracts possess a significant anti-AChE activity, which allows us to suggest that the polar solvent system is effectively able to extract better natural AChE inhibitor drugs. Similar to our findings, Gali and Bedjou [2] affirmed that alcoholic extracts of Algerian *R. chalepensis*, especially the n-butanol and EtOH extracts, exhibited a more significant inhibitory effect on AChE than organic fractions, with respective IC_50_ values of 67.94 ± 0.5 and 86.19 ± 3.6 μg/mL, while the aqueous extracts have been inactive. In contract, the MeOH extract of *R. graveolens* revealed a moderate inhibition capacity with an IC_50_ > 200 μg/mL (Talić et al., 2014) [68]. Compared to their results, the studied extracts of *R. tuberculata* seem to be the most active against AChE, and thus presented the lowest IC_50_ values.

Our results allow us to elicit the same suggestions of Eissa et al. [17], who reported that *H. tuberculatum* (or *R. tuberculata*) may have promising therapeutic proprieties, especially to prevent various neurodegenerative disorders, including Alzheimer and Parkinson’s diseases. Furthermore, the important anti-AChE activity expressed by *R. tuberculata* extracts, in particular by RME, could be attributed to their phenolic compounds. Indeed, several studies underlined that various phenolic acids with different structural and physicochemical characteristics may be displayed as AChE inhibitors, including gallic acid, noting that this phytocompound was exclusively identified in RAE and showed a remarkable anti-AChE activity (IC_50_ value of 5.85 μM). The antioxidant potential of this compound was also demonstrated [44,69]. Moreover, it was also reported that *p*-hydroxybenzoic acid [70] and some flavonoids such as rutin and quercetin [71], which were also detected in both studied extracts; revealed a noticeable AChE inhibitory activity with an IC_50_ values of 20.07, 86.0 and 62.0 μg/mL, respectively. In addition, the neuroprotective effect of sylimarin was demonstrated on various neurological diseases models, such as Alzheimer’s and Parkinson’s. Thus, its benefic effect is linked to its ability to reduce oxidative stress and inflammatory cytokines secretion, which may alter the cellular apoptosis via a neurodegenerative machinery process in the brain [10]. In addition, some studies reported that the most potent natural AChE inhibitors contained in plants are polar alkaloid and coumarin derivatives, which are easily extracted with polar solvents [72] and which may partially explain the excellent anti-AChE effect of *R. tuberculata* polar extracts due this plant’s richness in coumarins and various alkaloid derivatives [8,16].

One of the main characteristics of Alzheimer’s disease is the progressive deterioration of memory and intellectual capacities. Thus, the impairment of memory is present during the early stages of this disease. A study made by Hussein et al. [73] demonstrated that kaempferol can exert a significant neuroprotective effect against chlorpyrifos-induced oxidative stress. This flavonoid is also capable of exerting a noticeable general protection in Parkinson’s disease [74].

Vanillic acid could be also considered a potent neuroprotective agent, since this compound has the capacity to stimulate mitochondrial biogenesis in neuronal cells but also to control the expression of two key PARK genes, PARK2 and LRRK2, respectively [75]. Ullah et al. [76] also revealed that this phenolic acid has the capacity to prevent neuroinflammation, synaptic deficits and memory impairment by decreasing Aβ1-42-induced neuronal apoptosis, which was proved using HT22 cells [77].

It is also interesting to note that other phytocompounds such as p-Coumaric acid can exert a non-negligible regulatory effect on central cholinergic synapses, which in return will considerably improve cognitive and behavioral aptitudes, and this was proved using a scopolamine-induced learning and memory impairments model in the hippocampal zone of rats [78]. Sakamula and his collaborators also proved that this compound can prevent cerebral ischemia reperfusion injuries by increasing the activity of catalase and superoxide dismutase but also by reducing malondialdehyde (MDA) level, which considerably limited hippocampal neuronal death [79]. Cinnamic acid may also be considered a good candidate to prevent neurodegenerative diseases by reducing cerebral amyloid–beta plaque burden by activating the nuclear hormone receptor PPARα [80,81]. Note that this compound also helped in restoring synaptic concentrations of acetylcholine.

## 5. Conclusions

In conclusion, the present study indicated that the polar extraction of *R. tuberculata* showed remarkable antioxidant, anti-AChE, anti-arthritic, anti-inflammatory and antiulcer potential, by which they could be safely used for therapeutic purposes. Their pharmacological proprieties indicated that *R. tuberculata* aerial parts may provide a natural resource of alternative anticholinesterase able to prevent neurodegenerative diseases such as Alzheimer’s. Moreover, it could be considered as a source of natural antioxidants and anti-inflammatory and antiulcer compounds, which can treat various oxidative stress-related injuries and alleviate gastric ulcers. Further investigation should be required to explore the pharmacological efficiency of *R. tuberculata* against diseases.

## Figures and Tables

**Table 1:** Quantification of phenolic compounds in *R. tuberculata* extracts using cLC-DAD system.

N°	Compound	cLC-DAD			
		RT (min)	UV (nm)	RAE	RME
				Extracted Amount (mg/100 g d.E)	
**1**	**Gallic acid**	10.37	280	264.16	24.53
**2**	**Catechin**	14.12	280	7.00	16.75
**3**	**Chlorogenic acid**	Nt	Nt	Nt	Nt
**4**	**Gentisic acid**	Nt	Nt	Nt	Nt
**5**	***p*-hydroxybenzoic acid**	14.80	254	21.32	10.38
**6**	**Vanillic acid**	16.68	280	11.19	10.40
**7**	**Caffeic acid**	15.62	325	4.14	27.15
**8**	**4-hydroxy benzaldehyde**	Nt	Nt	Nt	Nt
**9**	***p*-coumaric acid**	17.32	280	2.41	2.93
**10**	**Trans-Ferulic acid**	17.94	325	10.28	3.19
**11**	**Rutin**	19.14	360	31.67	58.31
**12**	**Myricetin**	19.51	360	2624.82	5368.64
**13**	**Sylimarin**	19.96	280	701.62	700.00
**14**	**Naringenin**	18.02	280	20.21	69.97
**15**	**Quercetin**	20. 81	360	4.50	25.86
**17**	**Kaempferol**	Nt	Nt	Nt	Nt
**18**	**Cinnamic acid**	20. 89	280	25.38	100.99
**19**	**Trans-cinnamic acid**	21.36	280	112.07	131.87

RT: retention time; Nt: not tested.

**Table 2:** Antioxidant activities of the polar extracts from *R. tuberculata* aerial parts.

Extract/ Standard	Radical Scavenging		Lipid Peroxidation	Reducing Power		Total Antioxidant Capacity (TAC)
	DPPH	ABT’S		FRAP	CUPRAC	
	IC_50_ (μg/mL)			A_0.5_ (μg/mL)		AAE (mg/g E)
**RAE**	74,87 ± 1.1 ^ns,a,b,d^	143.54 ± 1.0 ^d^	254. 58 ± 1.7 ^d^	132.92 ± 0.9 ^ns,d^	196.9 ± 0.8 ^a,d^	369.57 ± 1.0 ^d^
**RME**	53.78 ± 0.5 ^ns,b,d^	80.72 ± 0.9 ^d^	153.18 ± 0.06 ^d^	132.71 ± 1.1 ^ns,d^	211.34 ± 2.2 ^d^	135.8 ± 0.02
**BHT**	49.77± 0.1 ^a,c,d^	1.55 ± 0.26 ^d^	1.24 ± 0.00 ^ns,d^	>50	9.62 ± 0.9 ^d^	Nt
**BHA**	15.74 ± 0.5 ^c,d^	7.54 ± 0.7 ^d^	1.26 ± 0.00 ^ns,d^	8.41 ± 0.7 ^a,d^	3.64 ± 0.2 ^d^	Nt
**Ascorbic acid**	26.38 ± 0.5 ^a,c,d^	Nt	Nt	9.01 ± 1.5 ^a,d^	Nt	Nt
**Gallic acid**	53.03 ± 0.0 ^b,c,d^	Nt	Nt	6.91 ± 0.0 ^a,d^	Nt	Nt
**Quercetin**	60.77 ± 0.0 ^a,d^	Nt	Nt	24.01 ± 0.0 ^d^	Nt	Nt

Values are expressed as means ± SD (*n* = 3) and are presented as ascorbic acid equivalent, IC_50_ and/or A_0.5_ values. Mean values followed by different letters are significantly different (based on one-way ANOVA followed by Tukey’s multiple comparison tests) ^ns^ is not significant, Level of Significance ^a^ *p* < 0.05, ^b^ *p* < 0.01, ^c^ *p* < 0.001, ^d^ *p* < 0.0001); nt: not tested.

**Table 3:** Inhibitory effect of *R. tuberculata* extracts on acetyl-cholinesterase and bovine albumin denaturation assays.

Extract/ Standard	AChE Inhibition Assay		Bovine Albumin Denaturation Assay	
	Max Inhibition (%)	IC_50_ (μg/mL)	Max Inhibition (%)	IC_50_ (μg/mL)
**RAE**	89.7 ± 1.5	51.08 ± 1.6 ^a,d^	57.9 ± 0.04	212.27 ± 0.04 ^b,c^
**RME**	82.3 ± 1.4	56.7 ± 0.97 ^a,d^	48.1 ± 0.02	>400
**Galanthamine**	94.7 ± 0.3	6.27 ± 1.1	Nt	Nt
**Diclofenac sodium**	Nt	Nt	84.9 ± 0.8	115.76 ± 0.8

Values are expressed as means ± SD (*n* = 3). Mean values followed by different letters are significantly different (based on one-way ANOVA followed by Tukey’s multiple comparison tests). Level of Significance ^a^ *p* < 0.05, ^b^ *p* < 0.01, ^c^ *p* < 0.001, ^d^ *p* < 0.0001); Nt: not tested.

**Table 4:** Anti-inflammatory activity of *R. tuberculata* polar extracts and indomethacin on Carrageenan-induced paw edema in mice.

Treatment	Dose (mg/kg, p.o.)	∆ Paw Diameter (mm)					
		Before Treatment	After Treatment				
			1 h	2 h	3 h	4 h	5 h
**Carrageenan-IPE Control**	**Normal Saline**	1.65 ± 0.02	2.82 ± 0.03	3.24 ± 0.03	3.68 ± 0.06	4.02 ± 0.01	4.37 ± 0.04
**Indomethacin**	20	1.62 ± 0.09 ^ns^	2,01 ± 0,07 ^c^ (28.73%)	2,00 ± 0,06 ^c^ (38.36%)	1,86 ± 0,04 ^d^ (49.64%)	1,76 ± 0,02 ^d^ (56.28%)	1.73 ± 0.04 ^d^ (60.37%)
**RAE**	200	1.67 ± 0.16 ^ns^	2.44 ± 0.12 ^b^ (13.55%)	2.24 ± 0.11 ^c^ (30.84%)	2.06 ± 0.12 ^c,a^ (43.98%)	1.99 ± 0.11 ^d,b^ (50.39%)	1.87 ± 0.07 ^d^ (57.16%)
**RAE**	400	1.65 ± 0.11 ^ns^	2.37 ± 0.05 ^b^ (15.92%)	2.19 ± 0.06 ^c^ (32.34%)	2.05 ± 0.04 ^c,a^ (44.48%)	1.96 ± 0.06 ^d,b^ (51.32%)	1.73 ± 0.02 ^d^ (60.32%)
**RME**	200	1.70 ± 0.07 ^ns^	2,43 ± 0.10 ^b^ (13.73%)	2,20 ± 0.17 ^c^ (31.95%)	2,17 ± 0.18 ^c,a^ (41.22%)	1.99 ± 0.17 ^d,b^(50.60%)	1.77 ± 0.02 ^d^ (59.45%)
**RME**	400	1.66 ± 0.11 ^ns^	2.35 ± 0.10 ^b^ (16.50%)	2.13 ± 0.11 ^c^ (34.09%)	2.02 ± 0.07 ^c,a^ (45.11%)	1.95 ± 0.08 ^d,b^ (51.51%)	1.67 ± 0.04 ^d^ (61.78%)

Carrageenan-IPE: Carrageenan-induced paw edema. Percentage inhibition of paw edema was expressed as mean ± SEM (*n* = 6). Mean values followed by different letters are significantly different (based on one-way ANOVA followed by Tukey’s multiple comparison tests, ^ns^ is no significant, Level of Significance: ^a^ *p* < 0.05, ^b^ *p* < 0.01, ^c^ *p* < 0.001, ^d^ *p* < 0.0001) compared to control groups.

**Table 5:** Anti-ulcerogenic activity of *R. tuberculata* polar extracts on ethanol-induced ulcers in mice.

Groups	Treatment	Dose (mg/kg)	Ulcer Index (UI)	PP (%)
**1**	EtOH-IGU Control	Normal saline (10 mL/kg)	4.7 ± 0.24	0
**2**	Omeprazole	20	0.9 ± 0.1 ^d^	80.9 ± 0.7
**3**	RAE	200	1.9 ± 0.12 ^c,b,a^	59.6 ± 0.63 ^c,a^
**4**	RAE	400	0.9 ± 0.05 ^d^	81.3 ± 0.6 ^ns,a^
**5**	RME	200	2.2 ± 0.13 ^b,c,a^	53.2 ± 1.3 ^c,a^
**6**	RME	400	1.1 ± 0.1 ^d^	76.6 ± 1.0 ^b^

EtOH-IGU: ethanol-induced gastric ulcers. Ulcer index (UI) and preventive percentage (PP) were expressed as mean ± SEM (*n* = 6). Mean values followed by different letters are significantly different (based on one-way ANOVA followed by Tukey’s multiple comparison tests, Level of Significance ^a^ *p* < 0.05, ^b^ *p* < 0.01, ^c^ *p* < 0.001, ^d^ *p* < 0.0001).

## Data Availability

Data is contained within the article.
